# Comparison of different reference values for lung function: implications of inconsistent use among centers

**DOI:** 10.1186/s12890-023-02430-7

**Published:** 2023-04-24

**Authors:** Henrik Mangseth, Liv Ingunn Bjoner Sikkeland, Michael Thomas Durheim, Mariann Ulvestad, Ole Henrik Myrdal, Johny Kongerud, May B Lund

**Affiliations:** 1 Department of Respiratory Medicine, Oslo University Hospital, Sognsvannsveien, Rikshospitalet, Oslo 20,0372 Norway; 2 Institute of Clinical Medicine, University of Oslo, Oslo, Norway

**Keywords:** Lung function tests, Reference values, Interpretation, Clinical aspects

## Abstract

**Background:**

For interpretation of pulmonary function tests (PFTs), reference values based on sex, age, height and ethnicity are needed. In Norway, the European Coal and Steel Community (ECSC) reference values remain widely used, in spite of recommendations to implement the more recent Global Lung Function Initiative (GLI) reference values.

**Objective:**

To assess the effects of changing from ECSC to GLI reference values for spirometry, DLCO and static lung volumes, using a clinical cohort of adults with a broad range in age and lung function.

**Methods:**

PFTs from 577 adults (18–85 years, 45% females) included in recent clinical studies were used to compare ECSC and GLI reference values for FVC, FEV1, DLCO, TLC and RV. Percent predicted and lower limit of normal (LLN) were calculated. Bland-Altman plots were used to assess agreement between GLI and ECSC % predicted values.

**Results:**

In both sexes, GLI % predicted values were lower for FVC and FEV1, and higher for DLCO and RV, compared to ECSC. The disagreement was most pronounced in females, with mean (SD) difference 15 (5) percent points (pp) for DLCO and 17 (9) pp for RV (p < 0.001). With GLI, DLCO was below LLN in 23% of the females, with ECSC in 49% of the females.

**Conclusions:**

The observed differences between GLI and ECSC reference values are likely to entail significant consequences with respect to criteria for diagnostics and treatment, health care benefits and inclusion in clinical trials. To ensure equity of care, the same reference values should be consistently implemented across centers nationwide.

**Supplementary Information:**

The online version contains supplementary material available at 10.1186/s12890-023-02430-7.

## Introduction

For interpretation of pulmonary function tests (PFTs), reference values based on sex, age, height and ethnicity are needed. Until 2012, the reference equations of choice in most European countries were those from the European Coal and Steel Community (ECSC), published as an official statement by the European Respiratory Society (ERS) in 1993 [1, 2]. In 2012, the Global Lung Function Initiative (GLI) published new reference equations for spirometry, based on tests from more than 97 000 multi-ethnic individuals, 3–95 years old [3]. In 2017 and 2021, GLI reference values for gas diffusion capacity for carbon monoxide (DLCO) and static lung volumes became available, based on tests from, respectively, more than 12,000 and 7000 individuals, 5–85/80 years old [4, 5].

The GLI reference values have been endorsed by all major respiratory societies, and disseminated to countries worldwide [6]. In 2018, Belgium was the first country to formally implement the GLI reference equations at a national level [7] and a recent study documented that the three sets of GLI reference values satisfactorily describe the lung function of pulmonary healthy Belgian adults [8]. In 2016, a Norwegian study demonstrated that the GLI-2012 reference values for spirometry fit population data satisfactorily, and were therefore recommended for nationwide use [9]. However, an informal telephone survey conducted prior to the annual meeting of the Norwegian Respiratory Society in 2021 showed that only half of the hospitals in the country had adhered to the recommendations.

Consistent interpretation of PFTs across centers is important to ensure equity of care. Percent predicted values for PFTs are often included in criteria for diagnosis and severity of pulmonary diseases, in guidelines for treatment, inclusion criteria for clinical studies, and legal assessment for health benefits [10–17]. Further, since the lower limit of normal (LLN) will vary with different sets of reference equations, the ability to discriminate health from disease may be affected. The PFTs for a given person may be above LLN using one equation while being below using another.

Several studies have examined the impact of changing from ECSC to GLI reference equations for spirometry [9, 18–23] while only a few have evaluated the effects for DLCO and static lung volumes [11, 24–26]. In the present study, we aimed to assess the effects of changing from ECSC to GLI reference equations on the interpretation of spirometry, DLCO and static lung volumes in a clinical dataset from a nationwide cohort of adults with a broad range of age and lung function.

## Materials and methods

### Design and study population

The study was conducted at the Department of Respiratory Medicine, Oslo University hospital, Rikshospitalet, Norway, a tertiary center with nationwide responsibilities. PFTs from 577 adult individuals, aged 18–85 years, all of Caucasian ethnicity, were included in the study. The PFTs were obtained from subjects who had participated in recent clinical studies [13, 27–31] and comprised four different groups (long-term survivors of severe blood disorders, n = 229; patients with pulmonary fibrosis, n = 148; lung transplant recipients, n = 88; and healthy, never-smoking controls, n = 112). The clinical studies had all been approved by the hospital’s Data Protection Officer and the Regional Committee for Medical and Health Research Ethics.

### Pulmonary function tests

The tests included dynamic spirometry, DLCO and determination of static lung volumes by whole body plethysmography. Registered variables were forced vital capacity (FVC), forced expiratory volume in one second (FEV1), DLCO, total lung capacity (TLC) and residual volume (RV). All tests were performed in accordance with the guidelines from ATS/ERS [32, 33]. Spirometry was performed without bronchodilator. Only tests from subjects with complete datasets that fulfilled the criteria for quality and acceptability were included. All PFTs were conducted in the same laboratory using Jaeger Master Screen Body (Eric Jaeger, Würzburg, Germany). Experienced physiologists, MSc, dedicated to the clinical studies and supervised by senior pulmonologists carried out the testing.

### Statistical analysis

All data from the PFTs were normally distributed, and are presented as mean, standard deviation (SD) or 95% confidence interval (CI), as appropriate. Predicted values were calculated using ERS’ reference calculator for GLI [34] and reference equations from ECSC [1, 2]. Lower limit of normal (LLN) was defined as the 5th percentile and corresponds to a Z-score of -1.645. To define airways obstruction and restriction, we used the ATS/ERS Task Force recommendations: An obstructive ventilatory impairment is defined by FEV1/FVC (or VC) below the LLN, which is defined as the 5th percentile of a normal population, and a restrictive ventilatory impairment is a reduction in TLC below the LLN (5th percentile) [35]. Bland-Altman plots were used to assess agreement between GLI and ECSC predicted values. Intra class correlation coefficients (ICC) were also calculated and displayed. Paired-sample t-test was used to analyze differences in % predicted between the two sets of reference values. Statistical significance was set as two-sided *p* < 0.05 [36]. A minimal difference of > 5% points (pp) in mean % predicted values between GLI and ECSC was defined as clinically significant. Prism v8.3.0 (GraphPad) was used for the Bland-Altman plots and other graphs, and SPSS (version 28, IBM) for all other statistical analyses.

## Results

**Fig. 1:**
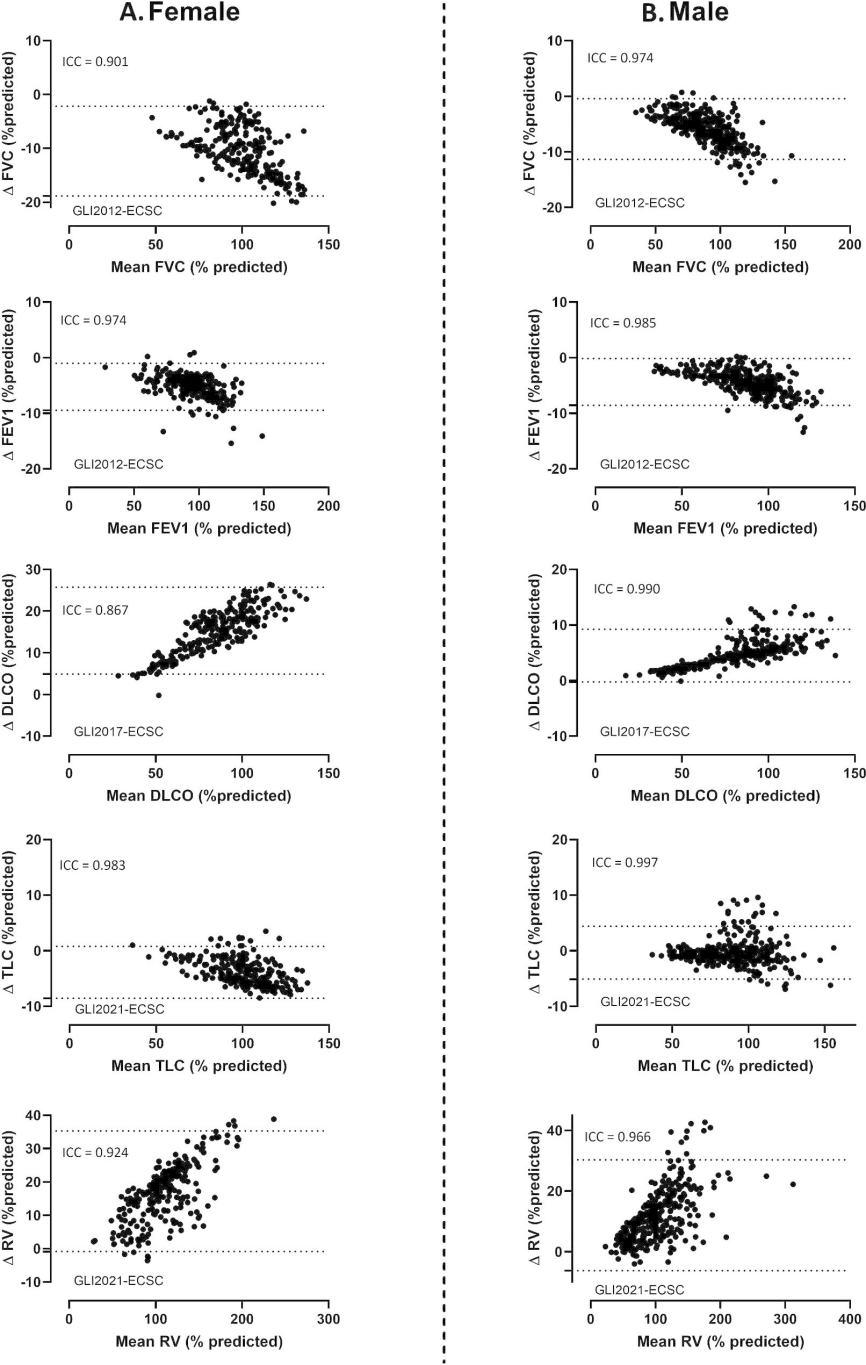
Bland-Altman plots of the measured values expressed as % predicted for FVC, FEV1, DLCO, TLC and RV, according to GLI and ECSC. X-axis: mean GLI and ESCS % predicted values. Y-axis: difference between % predicted GLI and %predicted ECSC. Panel A: Female, Panel B: Male. For each panel, the ICC is also displayed

Age and absolute values for all lung function variables, by sex and clinical groups, are outlined in Table 1. Mean (SD) height, weight and BMI were 167 (7) cm, 69 (14) kg and 25 (5) kg/m² for females and 180 (7) cm, 85 (15) kg and 26 (4) kg/m² for males, and did not differ significantly between the clinical groups. Figure 1 shows Bland-Altman plots for overall agreement between GLI and ECSC for % predicted values for FVC, FEV1, DLCO, TLC and RV, stratified by sex. For both sexes, the GLI % predicted values for TLC aligned well with the ECSC % predicted values. The largest differences between GLI and ECSC % predicted values were observed for RV and DLCO, and especially in females. For FVC and FEV1 the differences between GLI and ECSC were below zero; i.e. the % predicted values were lower with GLI than with ECSC. The opposite trend was seen for DLCO and RV; i.e. GLI equations gave higher % predicted values than ECSC. ICC scores are also displayed on the plots, for each lung function variable.

**Fig. 2:**
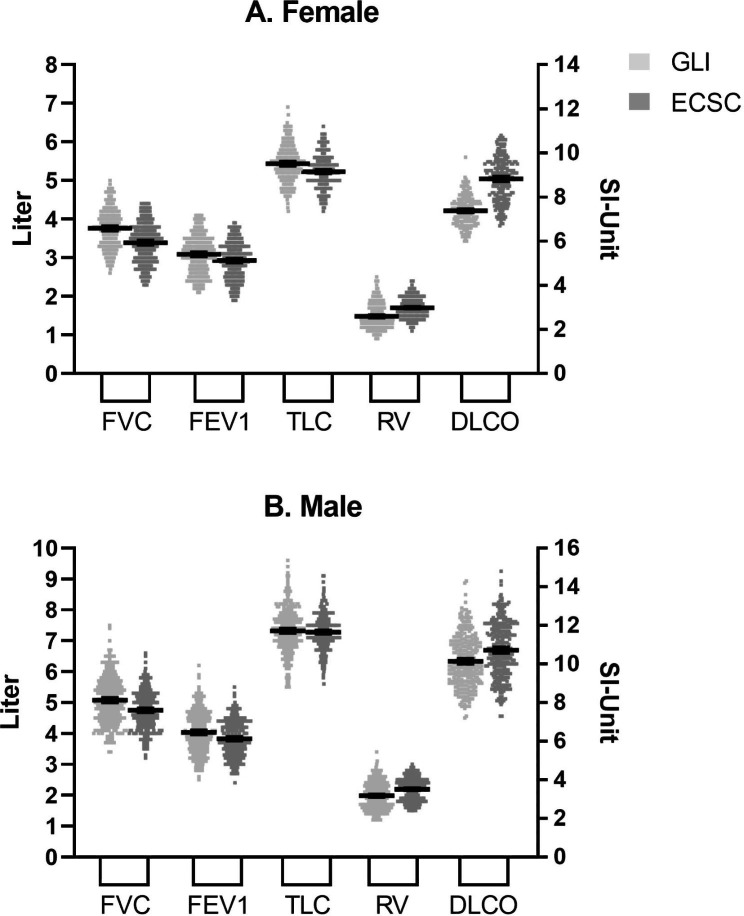
Scatter plots (mean and 95% CI) between predicted values in original units for FVC, FEV1, DLCO, TLC and RV, according to GLI (light grey square) and ECSC (dark grey square). Left y-axis: Liters. Right y-axis: SI-unit (mmol/min*kPa). Panel A: Female, Panel B: Male. p < 0.001 for all variables except TLC for male

Figure 2 shows scatter plots (mean, 95% CI) for predicted values in original units for FVC, FEV1, DLCO, TLC and RV, according to GLI and ECSC reference equations. In both sexes, GLI gave significantly higher reference value for FVC, FEV1, and lower for DLCO and RV.

**Fig. 3:**
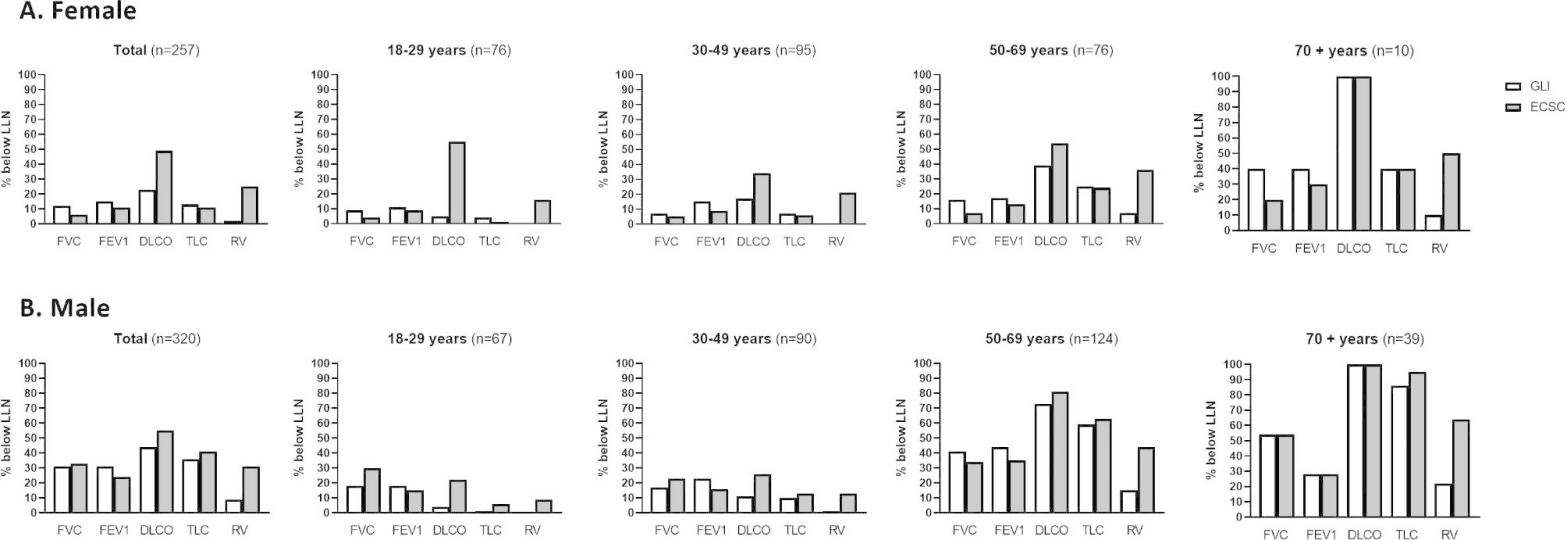
Patients (%) with values below the lower limit of normal (LLN), according to sex and age groups, for FVC, FEV1, DLCO, TLC and RV. White columns: GLI, grey columns: ECSC. Panel A: Female, Panel B: Male

The observed differences between GLI and ECSC reference values also affect the number of individuals that fall below LLN. Figure 3 shows the proportions of subjects with PFTs below LNN, by age-groups and sex. In our population, 23% of the females were below LLN in DLCO using GLI, while 49% were below with ECSC (p < 0.001). The difference was most pronounced in younger females. This means that by changing from ECSC to GLI reference equations, one fourth of the females would go from below to above LLN, hence reclassified from ‘sick’ to ‘healthy’ if LLN were used to dichotomize. In males the largest difference between GLI and ECSC for LLN was observed in RV, particularly in the older groups (age 50–85 years) where 16% would fall below LLN with GLI and 49% with ECSC.

In additional document, table S1 provides detailed information on mean (SD) predicted FVC, FEV1, DLCO, TLC and RV (in absolute values and % predicted) and LLN, using both GLI and ECSC reference equations, by sex and age groups.

## Discussion

The present study reports the impact of changing from ECSC to GLI reference equations for PFTs in a nationwide adult clinical population. The main findings were that GLI equations gave lower % predicted values for FVC and FEV1, and higher % predicted values for DLCO and RV, and that these differences were of clinical significance. The differences were most pronounced in females. In sum, the findings underline the importance of using the same reference equations across centers in order to obtain consistent interpretation of PFTs and thereby ensure equity in health care.

A previous study has shown that the GLI reference values for spirometry fit Norwegian population data better than the ECSC reference values [9]. In line with that study, we found significantly lower predicted values for FVC and FEV1 with GLI than with ECSC. For static lung volumes, we found that the GLI equations for TLC aligned well with the ECSC equations, while GLI equations gave significantly higher % predicted RV than those from ECSC. For DLCO, we found significantly higher predicted values with GLI than with ECSC, which is in accordance with reports from a French study that also included a mixed clinical population [25]. Regrettably, there are no appropriate Norwegian population data on static lung volumes or DLCO that can be used to determine whether GLI or ECSC will give the best fit. The only available data on DLCO dates from the 1980s and comprised tests from 304 subjects, aged 18–73 years [37]. For comparison, the GLI reference values ​​for DLCO are based on data from 10,765 Caucasians aged 4½-85 years. However, since it has been well documented that the GLI equations for spirometry fit the Norwegian population data better than the ECSC equations, it is not unreasonable to speculate that the GLI equations for DLCO and static lung volumes would also provide the best fit.

As far as we know, only two large clinical studies have compared GLI and ECSC reference values ​​for DLCO [24, 25]. A French study compared data from 4180 DLCO tests obtained from 2898 adults, including both healthy subjects and patients with various pulmonary disorders [25]. They found that GLI gave higher % of predicted DLCO than ECSC in both males and females, but the difference was greatest in females. Another study, from Australia, included DLCO measurements from a non-selected patient population (n = 33,863) aged 5–85 years, registered during the period 2008–2018 [24]. That study also reported higher % predicted DCLO with GLI than with ECSC, but the most important finding was that fewer patients fell below LLN when switching from ECSC to GLI, especially among young females. The results from both the French and the Australian studies support our findings. However, one must bear in mind that the number of subjects in a study population that will fall below LLN depends not only on the choice of reference equations, but also on the composition of the population. In our study population, comparison of the use of GLI and ECSC resulted in 23% vs. 49% of the females having a DLCO below LLN. The difference was largest in the youngest age group, where 55% were below LNN with ECSC and only 5% with GLI. In the oldest age group, more than 90% of the subjects fell below LLN with both ECSC and GLI equations. The likely explanation for this is that the elderly portion of our study population primarily comprised patients with pulmonary fibrosis.

While the ECSC equations required extrapolation for subjects older than 70 years, the GLI reference population includes subjects up to 95 years of age for spirometry, 85 years for DLCO and 80 years for static lung volumes. A Portuguese geriatric study of 260 subjects aged 65–95 years documented that the differences between the ECSC and the GLI reference equations for spirometry ​​also apply to an elderly population [19]. A rapidly increasing aging population, as well as the increasing incidence of lung disorders with increasing age, lends further support to replacing ECSC with GLI reference values - unless national population data suggest otherwise.

The use of different reference values ​​within one country may have various clinical implications. Most importantly, % predicted values for PFTs ​​are often used as cut-off criteria for diagnosis of pulmonary disease, severity of disease and indication for therapy. In the Global Initiative for Chronic Obstructive Lung Disease (GOLD), severity of the disease is categorized into stages based on % predicted FEV1% (mild ≥ 80%, moderate 79 − 50%, severe 49 − 30% and very severe < 30%) [38]. In our study, mean % predicted FEV1 was five pp lower with GLI equations than with ECSC, which means that for some patients the choice of reference values may affect the grading of severity of COPD. In total, we identified obstructive ventilatory defects in 13% with GLI and 10% with ECSC, and the difference between GLI and ECSC was largest in males (16% vs. 12%). These findings are supported by other studies [18–20]. The composition of the study population is important to bear in mind. Our study did not include a specific clinical group with COPD. The subjects with airways obstruction were primarily diagnosed with bronchiolitis obliterans syndrome, secondary to lung transplantation or hematopoietic stem cell transplantation.

Measurements of static lung volumes are used to define both hyperinflation and restrictive impairment [39]. In studies of therapeutic interventions for emphysema, cut-off values for % predicted TLC and RV are commonly used as criteria for inclusion. TLC > 100% predicted and RV > 150% predicted have been used as criteria for inclusion in studies of, volume reduction surgery [15] and volume reduction by endobronchial valves [16], respectively.

In clinical pharmacological trials, the criteria for inclusion and exclusion will usually include cut-off values for % predicted PFTs. A Dutch study examined the effect of changing from ECSC to GLI reference values with respect to inclusion in the trial of a new drug against idiopathic pulmonary fibrosis [11]. DLCO 30% predicted was the lower limit for inclusion, and by switching from ECSC to GLI reference values, several more patients met the requirement and might have been allowed to participate in the study.

When persons with work-related lung disorders claim compensation from public healthcare systems or private health insurance, the level of compensation for health loss will depend on the degree of disability. For occupational lung diseases, % predicted PFTs will invariably be used to determine the severity of disease and the degree of disability [17]. The higher the degree of disability, the higher the compensation. Therefore, choice of reference values may entail financial consequences, both for workers entitled to compensation and for the health insurance systems.

The main strengths of the present study were that all PFTs were performed at the same laboratory and with the same equipment. Further, in order to ensure optimal data quality and feel confident that the ERS/ATS guidelines had been strictly followed, we opted to use PFTs that had been obtained in the context of research projects, instead of unselected PFTs from the daily clinical routine. All testing was carried out by experienced respiratory physiologists, specifically dedicated to the research projects. Our hospital is a tertiary university center with national responsibilities, and the study population comprised subjects from all parts of the country. Therefore, the results of the study may be generalized nationwide. However, the study also has limitations. Most importantly, the PFTs were obtained from adults only. Pulmonary function testing is also frequently carried out in children, and the reference values ​​from GLI include children as young as 4–5 years of age. Research data assessing the fit of GLI equations in various pediatric populations across nations are scarce [20, 24, 40, 41], and more studies are warranted.

## Conclusion

Inconsistent use of GLI and ECSC reference equations for PFTs across different centers may have clinical consequences that affect the criteria for diagnosis and severity of disease, eligibility for health care benefits, and inclusion in clinical trials. In order to ensure consistent interpretation of PFTs, the same reference values should be consistently used across centers nationwide. Since ERS has endorsed the GLI reference values, we encourage all centers that still use the old ECSC equations to update to GLI.

**Table 1:** Characteristics of the study population, according to clinical groups, and stratified by sex

	Total (n = 577)		Healthy (n = 112)		Survivors of blood disorders (n = 229)		Lung fibrosis (n = 148)		Lung TX (n = 88)		
	**Female** (n = 257)	**Male** (n = 320)	**Female** (n = 67)	**Male** (n = 45)	**Female** (n = 118)	**Male** (n = 111)	**Female** (n = 33)	**Male** (n = 115)	**Female** (n = 39)	**Male** (n = 49)	
**Age**	42 (16)	48 (18)	42 (15)	39 (11)	33 (10)	32 (109	66 (6)	65 (9)	52 (13)	55 (9)	
**FVC** (L)	3.6 (0.8)	4.4 (1.3)	4.0 (0.6)	5.8 (0.8)	3.8 (0.6)	5.2 (1.0)	2.4 (0.6)	3.2 (0.7)	3.3 (0.7)	3.9 (1.0)	
**FEV1** (L)	2.9 (0.7)	3.4 (1.1)	3.2 (0.6)	4.5 (0.7)	3.0 (0.6)	4.1 (0.8)	1.9 (0.5)	2.6 (0.5)	2.5 (0.6)	2.7 (0.9)	
**FEV1/FVC**	0.80 (0.08)	0.78 (0.10)	0.79 (0.07)	0.77 (0.06)	0.81 (0.08)	0.79 (0.09)	0.79 (0.07)	0.80 (0.07)	0.79 (0.11)	0.69 ﻿(0.16)	
**DLCO** (SI-unit)	6.9 (2)	8.2 (3.4)	8.1 (1.3)	11.6 (1.8)	7.5 (1.4)	10.9 (2.0)	3.8 (0.8)	4.9 (1.3)	5.6 (1.5)	7.0 (1.8)	
**TLC** (L)	5.3 (1)	6.5 (1.7)	6.1 (0.7)	8.3 (1.1)	5.4 (0.8)	7.3 (1.4)	4.0 (1.0)	5.1 (1.0)	5.0 (1.0)	6.4 (1.3)	
**RV** (L)	1.7 (0.6)	2.1 (0.8)	2.0 (0.5)	2.5 (0.6)	1.6 (0.5)	2.1 (0.7)	1.5 (0.5)	1.8 (0.6)	1.7 (0.6)	2.5 (1.3)	

TX = transplantation. Data as mean (SD). SI-unit = mmol/min*kPa

## Electronic supplementary material

Below is the link to the electronic supplementary material.

Supplementary Material 1

## Data Availability

All data generated and analyzed during this study are included in this article. Further enquiries can be directed to the corresponding author.

## Abbreviations

ATS: American Thoracic Society
CI: Confidence interval
COPD: Chronic obstructive pulmonary disease
DLCO: Gas diffusion capacity for carbon monoxide
ECSC: European Coal and Steel Community
ERS: European Respiratory Society
FEV1: Forced expiratory volume in 1 s
FVC: Forced vital capacity
GLI: Global Lung Initiative
ICC: Intraclass correlation coefficients
LLN: Lower limit of normality
PFT: Pulmonary function test
RV: Residual volume
SD: Standard deviation
TLC: Total lung capacity
TX: Transplantation
