# Supplementary material for: Comparison of different reference values for lung function: implications of inconsistent use among centers
Source: BMC Pulm Med. 2023 Apr 24;23:137. doi: 10.1186/s12890-023-02430-7 (PMC10127329; doi:10.1186/s12890-023-02430-7)
Supplement: Supplementary file 1 — Supplementary Material 1 [file 12890_2023_2430_MOESM1_ESM.docx]

| **Table S1**. Comparison of GLI and ECSC in absolute- and predicted values, %predicted, and difference in %predicted for spirometry, DLCO and static lung volumes stratified by sex and age groups | | | | | | | | | | | | | | | | | |
| --- | --- | --- | --- | --- | --- | --- | --- | --- | --- | --- | --- | --- | --- | --- | --- | --- | --- |
| **A. Female** | |  |  | **Age groups** | | | |  | **B. Male** | |  |  | **Age groups** | | | |  |
|  |  |  | **Total** (n=257) | **18-29** (n=76) | **30-49** (n=95) | **50-69** (n=76) | **70 +** (n=10) |  |  |  |  | **Total** (n=320) | **18-29** (n=67) | **30-49** (n=90) | **50-69** (n=124) | **70 +** (n=39) |  |
| **FVC** |  | Liter | 3.6 (0.8) | 3.9 (0.7) | 3.8 (0.6) | 3.1 (0.8) | 2.5 (0.9) |  | **FVC** |  | Liter | 4.4 (1.3) | 5.3 (1.1) | 5.3 (1.1) | 3.7 (0.9) | 3 (0.7) |  |
|  | GLI | Predicted values (L) | 3.8 (0.5) | 4.1 (0.4) | 3.9 (0.3) | 3.3 (0.3) | 3 (0.2) |  |  | GLI | Predicted values (L) | 5.1 (0.7) | 5.6 (0.6) | 5.5 (0.6) | 4.7 (0.5) | 4.2 (0.4) |  |
|  |  | LLN (L) | 3.0 (0.5) | 3.3 (0.3) | 3.1 (0.3) | 2.5 (0.3) | 2.2 (0.2) |  |  |  | LLN (L) | 4.0 (0.7) | 4.6 (0.5) | 4.4 (0.5) | 3.6 (0.4) | 3.1 (0.3) |  |
|  |  | % predicted | 95 (16) | 95.5 (12.3) | 96.5 (14.1) | 94.4 (19.9) | 81.9 (24.1) |  |  |  | % predicted | 85 (19) | 94.1 (13.1) | 95.2 (17.8) | 77.7 (17.5) | 71.5 (14.1) |  |
|  | ECSC | Predicted values (L) | 3.4 (0.5) | 3.9 (0.3) | 3.5 (0.3) | 2.9 (0.3) | 2.7 (0.2) |  |  | ECSC | Predicted values (L) | 4.7 (0.6) | 5.3 (0.5) | 5.1 (0.5) | 4.4 (0.4) | 4.0 (0.4) |  |
|  |  | LLN (L) | 2.7 (0.5) | 3.2 (0.3) | 2.8 (0.3) | 2.2 (0.3) | 2 (0.2) |  |  |  | LLN (L) | 4.0 (0.9) | 4.9 (0.7) | 4.4 (0.8) | 3.4 (0.4) | 3.0 (0.4) |  |
|  |  | % predicted | 106 (19) | 101.3 (13.3) | 108.3 (16.2) | 108.1 (22.7) | 91.6 (26.3) |  |  |  | % predicted | 91 (21) | 99.7 (14.8) | 103.4 (19.4) | 83.3 (18.9) | 73.8 (14.6) |  |
|  | ΔGLI-ECSC | Difference %pred | 11 (4) | 5.8 (2.6) | 11.8 (2.8) | 13.7 (3.1) | 9.7 (2.7) |  |  | ΔGLI-ECSC | Difference %pred | 6 (3) | 5.6 (3) | 8.2 (2.4) | 5.5 (1.8) | 2.3 (0.9) |  |
| **FEV1** |  | Liter | 2.9 (0.7) | 3.3 (0.5) | 3 (0.6) | 2.4 (0.6) | 1.9 (0.6) |  | **FEV1** |  | Liter | 3.4 (1.1) | 4.3 (0.9) | 4 (0.9) | 2.8 (0.8) | 2.4 (0.5) |  |
|  | GLI | Predicted values (L) | 3.1 (0.5) | 3.5 (0.3) | 3.2 (0.3) | 2.6 (0.3) | 2.3 (0.2) |  |  | GLI | Predicted values (L) | 4.0 (0.79 | 4.7 (0.5) | 4.4 (0.5) | 3.7 (0.4) | 3.1 (0.3) |  |
|  |  | LLN (L) | 2.4 (0.4) | 2.8 (0.3) | 2.6 (0.2) | 2.0 (0.2) | 1.7 (0.1) |  |  |  | LLN (L) | 3.1 (0.6) | 3.8 (0.4) | 3.5 (0.4) | 2.8 (0.3) | 2.2 (0.2) |  |
|  |  | % predicted | 92 (17) | 93.3 (13.1) | 93.2 (16.5) | 91.7 (20.3) | 81.3 (20.2) |  |  |  | % predicted | 83 (19) | 90.2 (15.6) | 90.1 (17.7) | 76.2 (19.1) | 77.7 (15.3) |  |
|  | ECSC | Predicted values (L) | 2.9 (0.5) | 3.4 (0.3) | 3.0 (0.3) | 2.4 (0.3) | 2.3 (0.2) |  |  | ECSC | Predicted values (L) | 3.8 (0.6) | 4.5 (0.3) | 4.2 (0.4) | 3.5 (0.3) | 3.1 (0.3) |  |
|  |  | LLN (L) | 2.3 (0.5) | 2.8 (0.3) | 2.4 (0.3) | 1.8 (0.3) | 1.6 (0.2) |  |  |  | LLN (L) | 3.0 (0.6) | 3.6 (0.3) | 3.3 (0.4) | 2.6 (0.3) | 2.2 (0.3) |  |
|  |  | % predicted | 98 (18) | 97.3 (13.4) | 99.1 (17.5) | 97.7 (21.8) | 83.6 (19.9) |  |  |  | % predicted | 88 (20) | 95 (16.8) | 95.8 (19) | 80.1 (20.1) | 79.4 (15.6) |  |
|  | ΔGLI-ECSC | Difference %pred | 5 (2) | 4 (1.4) | 5.9 (1.2) | 6 (2.7) | 2.3 (2.2) |  |  | ΔGLI-ECSC | Difference %pred | 4 (2) | 4.8 (2.5) | 5.7 (2) | 3.9 (1.3) | 1.8 (1) |  |
| **DLCO** |  | SI-unit | 6.9 (2) | 7.7 (1.4) | 7.5 (1.8) | 5.7 (1.7) | 3.4 (0.5) |  | **DLCO** |  | SI-unit | 8.2 (3.4) | 11.2 (2) | 10.7 (2.3) | 6.1 (2.1) | 4.2 (0.8) |  |
|  | GLI | Predicted values (SI) | 7.4 (0.7) | 7.8 (0.6) | 7.5 (0.5) | 6.8 (0.4) | 6.7 (0.3) |  |  | GLI | Predicted values (SI) | 10.1 (1.3) | 11.3 (1.1) | 10.9 (1) | 9.4 (0.7) | 8.6 (0.7) |  |
|  |  | LLN (SI) | 5.8 (0.6) | 6.2 (0.5) | 6.0 (0.4) | 5.2 (0.4) | 5.2 (0.4) |  |  |  | LLN (SI) | 7.8 (1.2) | 8.9 (0.9) | 8.5 (0.8) | 7.1 (0.6) | 6.3 (0.5) |  |
|  |  | % predicted | 93 (23) | 98.7 (15.5) | 100 (22.1) | 83.3 (23.4) | 51.5 (8.1) |  |  |  | % predicted | 7.8 (1.2) | 98.8 (14.2) | 98 (19.3) | 64.7 (20.2) | 48.8 (8.5) |  |
|  | ECSC | Predicted values (SI) | 8.8 (1.0) | 9.7 (0.6) | 9.0 (0.6) | 7.9 (0.6) | 7.5 (0.4) |  |  | ECSC | Predicted values (SI) | 10.7 (1.4) | 12.2 (0.9) | 11.5 (1) | 9.9 (0.8) | 8.9 (0.8) |  |
|  |  | LLN (SI) | 6.9 (1.0) | 7.8 (0.6) | 7.1 (0.6) | 5.9 (0.6) | 5.6 (0.4) |  |  |  | LLN (SI) | 8.4 (1.4) | 9.9 (0.9) | 9.2 (1) | 7.6 (0.8) | 6.6 (0.8) |  |
|  |  | % predicted | 77 (19) | 79.2 (12.4) | 83.5 (18.4) | 72.2 (19.9) | 45.9 (7.2) |  |  |  | % predicted | 75 (24) | 91.5 (13.4) | 92.9 (18.2) | 61.5 (19.1) | 47.2 (8.2) |  |
|  | ΔGLI-ECSC | Difference %pred | 15 (5) | 19.5 (3.7) | 16.4 (3.9) | 11 (3.6) | 5.6 (1) |  |  | ΔGLI-ECSC | Difference %pred | 5 (2) | 7.3 (2.6) | 5.2 (1.5) | 3.3 (1.1) | 1.9 (0.6) |  |
| **TLC** |  | Liter | 5.3 (1) | 5.4 (0.9) | 5.6 (0.8) | 5.1 (1.2) | 4.3 (1.3) |  | **TLC** |  | Liter | 6.5 (1.7) | 7.2 (1.3) | 7.6 (1.6) | 5.8 (1.4) | 4.8 (1) |  |
|  | GLI | Predicted values (L) | 5.4 (0.5) | 5.4 (0.6) | 5.6 (0.5) | 5.3 (0.4) | 5.4 (0.4) |  |  | GLI | Predicted values (L) | 7.3 (0.7) | 7.1 (0.8) | 7.5 (0.8) | 7.3 (0.6) | 7.3 (0.6) |  |
|  |  | LLN (L) | 4.4 (0.4) | 4.4 (0.5) | 4.5 (0.4) | 4.3 (0.4) | 4.3 (0.3) |  |  |  | LLN (L) | 5.9 (0.6) | 5.7 (0.7) | 6.1 (0.6) | 5.9 (0.5) | 5.7 (0.5) |  |
|  |  | % predicted | 98 (17) | 100.2 (12.2) | 101.4 (13.4) | 94.9 (21) | 78.7 (21.2) |  |  |  | % predicted | 89 (21) | 101.9 (12) | 101.3 (18.3) | 79.5 (17.6) | 67.1 (12.6) |  |
|  | ECSC | Predicted values (L) | 5.2 (0.4) | 5.3 (0.5) | 5.2 (0.4) | 5.1 (0.4) | 5.3 (0.4) |  |  | ECSC | Predicted values (L) | 7.3 (0.6) | 7.3 (0.6) | 7.4 (0.7) | 7.2 (0.5) | 7.2 (0.5) |  |
|  |  | LLN (L) | 4.2 (0.4) | 4.3 (0.5) | 4.3 (0.4) | 4.2 (0.4) | 4.4 (0.4) |  |  |  | LLN (L) | 6.1 (0.6) | 6.1 (0.6) | 6.3 (0.7) | 6.1 (0.5) | 6.1 (0.5) |  |
|  |  | % predicted | 102 (18) | 102.4 (13) | 107.3 (14.2) | 98.4 (22.2) | 79.3 (21.7) |  |  |  | % predicted | 89 (21) | 99.4 (12.9) | 102.7 (18.8) | 80.6 (18) | 66.6 (12.7) |  |
|  | ΔGLI-ECSC | Difference %pred | 4 (2) | 2.2 (2.3) | 5.9 (1) | 3.4 (1.7) | 0.6 (0.7) |  |  | ΔGLI-ECSC | Difference %pred | 0 (2) | 2.5 (3.2) | 1.4 (1.8) | 1.1 (1.1) | 0.2 (0.9) |  |
| **RV** |  | Liter | 1.7 (0.6) | 1.5 (0.4) | 1.8 (0.6) | 1.9 (0.6) | 1.7 (0.5) |  | **RV** |  | Liter | 2.1 (0.8) | 1.9 (0.6) | 2.3 (0.9) | 2.1 (0.8) | 1.9 (0.6) |  |
|  | GLI | Predicted values (L) | 1.5 (0.3) | 1.2 (0.1) | 1.4 (0.2) | 1.8 (0.2) | 2.2 (0.2) |  |  | GLI | Predicted values (L) | 2.0 (0.4) | 1.5 (0.2) | 1.7 (0.2) | 2.2 (0.2) | 2.7 (0.2) |  |
|  |  | LLN (L) | 0.8 (0.2) | 0.6 (0.1) | 0.8 (0.1) | 1.1 (0.1) | 1.3 (0.1) |  |  |  | LLN (L) | 1.1 (0.3) | 0.7 (0.1) | 0.9 (0.2) | 1.4 (0.1) | 1.6 (0.1) |  |
|  |  | % predicted | 120 (38) | 124.6 (29.9) | 131.8 (41.3) | 106.1 (34.1) | 80.1 (26.8) |  |  |  | % predicted | 110 (44) | 126.9 (34.1) | 135.7 (43.9) | 94.9 (38.3) | 70.5 (22.6) |  |
|  | ECSC | Predicted values (L) | 1.7 (0.3) | 1.5 (0.1) | 1.7 (0.1) | 2.0 (0.1) | 2.2 (0.1) |  |  | ECSC | Predicted values (L) | 2.2 (0.4) | 1.7 (0.1) | 2.0 (0.2) | 2.4 (0.1) | 2.8 (0.1) |  |
|  |  | LLN (L) | 1.1 (0.3) | 0.9 (0.1) | 1.1 (0.1) | 1.4 (0.1) | 1.6 (0.1) |  |  |  | LLN (L) | 1.5 (0.4) | 1.0 (0.1) | 1.3 (0.2) | 1.8 (0.1) | 2.1 (0.1) |  |
|  |  | % predicted | 103 (31) | 102.5 (24.9) | 110.2 (34.2) | 97.2 (30.6) | 78.5 (24.8) |  |  |  | % predicted | 98 (38) | 112.6 (31.1) | 115.7 (39.3) | 86.9 (34.8) | 67.3 (21.6) |  |
|  | ΔGLI-ECSC | Difference %pred | 17 (9) | 22.1 (5.8) | 21.6 (7.7) | 8.9 (5.4) | 1.7 (4.3) |  |  | ΔGLI-ECSC | Difference %pred | 12 (9) | 14.3 (7.2) | 19.9 (9.0) | 8.0 (6.2) | 2.0 (3.9) |  |
| Data as mean (SD). | | | | | | | | | | | | | | | | | |
